# A disulfidptosis-related lncRNA index predicting prognosis and the tumor microenvironment in colorectal cancer

**DOI:** 10.1038/s41598-023-47472-3

**Published:** 2023-11-16

**Authors:** Lijun Xiao, Wen Yin, Xuanqin Chen, Xu Zhang, Chao Zhang, Zehui Yu, Muhan Lü

**Affiliations:** 1 Department of Gastroenterology, The Affiliated Hospital of Southwest Medical University, Luzhou City, China; 2 The Affiliated Hospital of Southwest Medical University, Luzhou City, China; 3 Laboratory Animal Center, Southwest Medical University, Luzhou City, China; 4 Human Microecology and Precision Diagnosis and Treatment of Luzhou Key Laboratory, Luzhou City, China

**Keywords:** Cancer, Computational biology and bioinformatics, Diseases, Medical research

## Abstract

Colorectal cancer (CRC) is a common and deadly cancer worldwide with a high lethality rate. Disulfidptosis has been found to be an emerging mode of death in cancer, and the purpose of this study was to explore the relationship between disulfidptosis-related lncRNAs (DRLs) and CRC and to develop a prognostic model for CRC and DRLs. The gene expression data and clinicopathologic information of colorectal cancer patients were obtained from The Cancer Genome Atlas (TCGA) and screened for DRLs based on correlation analysis. The least absolute shrinkage and selection operator (LASSO) and Cox regression were used to construct the prognostic model, and its validation was carried out by PCA and receiver operating characteristic (ROC) curves. We constructed nomograms combined with the model. Finally, the possible mechanisms by which lncRNAs affect CRC were explored by functional enrichment analysis, immune infiltration and immune escape analysis. In summary, we developed a prognostic marker consisting of lncRNAs associated with disulfidptosis to help clinicians predict the survival of different CRC patients and use different targeted therapies and immunotherapies depending on the condition.

## Introduction

Colorectal cancer (CRC) is the third most common cancer in the world and has the second highest mortality rate of all cancers. Epidemiological statistics indicate that colorectal cancer affects more than 1.8 million people each year, resulting in approximately 900,000 deaths^1^. Colorectal cancer-related deaths account for 30.6% (286,162 out of 935,173) of all cancer-related deaths globally^2,3^. With the development of developing countries, the number of new cases of colorectal cancer is projected to increase to 2.5 million by 2035^4^. Early detection and correct prediction of prognosis can help physicians make correct clinical decisions and improve patient prognosis. The gold standard for tumor prognosis remains the tumor, lymph node, metastasis (TNM) staging system^5^. Previously, each feature of TNM staging of tumors has been widely used to assess patient survival and prognosis, given the heterogeneity of the disease and the limitations of the TNM system itself, which does not predict the prognosis of patients with different features and requires the development of more refined prediction models^6^. Therefore, the search for biomarkers with prognostic significance is particularly important for predicting overall or progression-free survival or recurrence rates, informing patients, and supporting proper medical decision-making.

Disulfidptosis is a mode of cell death that is independent of the current process of programmed death and is caused by the presence of excessive disulfide bonds within proteins. Previous studies have found that to counteract oxidative stress in unfavorable survival environments, tumor cells transport cystine intracellularly via high expression of the cystine transporter solute carrier family 7 member 11 (SLC7A11) to feed antioxidant molecules, such as GSH, a process that is highly dependent on NADPH produced by the pentose phosphate pathway^7^. Therefore, in glucose-deficient conditions, due to NADPH deficiency, cystine accumulates in large quantities in SLC7A11-overexpressing cells, and disulfide bonds are formed between sulfhydryl groups within cystine. The accumulation of large quantities of disulfide bonds leads to bisulfide stress, which results in changes in the structural function of actin proteins and destruction of the cytoskeleton and ultimately leads to programmed cell death, i.e., disulfidptosis^8^. It is not associated with ATP depletion or cystine crystal formation, is not blockable by other inhibitors of cell death, and cannot be prevented by knockdown of key ferroptosis/apoptosis genes^8^. Given the important role of various modes of cell death in tumor cells, further understanding of the expression patterns of disulfide bonds and the associated intrinsic mechanisms will help to identify potential therapeutic targets in colorectal cancer.

Long noncoding RNA (lncRNA) is an RNA molecule and an important component of the noncoding genome. Although most lncRNAs do not undergo translation, they participate in various complex biological processes^9^. Previous studies have shown that mutations and dysregulated expression of lncRNAs are associated with the development of cancer and that aberrant expression of lncRNAs disrupts normal cell growth and development, interferes with the cell cycle and metabolism, and leads to the development of various diseases^10^. LncRNAs have become key factors for a range of cancers, including colorectal cancer (CRC). However, to date, the specific lncRNAs and potential mechanisms that regulate disulfidptosis and affect CRC prognosis remain unclear. Exploring the abnormal expression of lncRNAs in CRC may reveal potential biomarkers and new therapeutic targets for CRC. Accurately predicting tumor prognosis and improving patient quality of life are the core goals of cancer treatment. Therefore, this study aimed to determine the prognostic role of lncRNAs related to disulfidptosis in colorectal cancer, identify new targets for biological therapies, develop a survival risk prediction model that can be used for prognostic prediction and selection of specific therapies for CRC patients, and uncover the mechanisms of disulfide rupture-induced cell death in colorectal cancer.

## Results

### Identification of disulfidptosis-related genes (DRGs) and lncRNAs (DRLs) in CRC

**Figure 1:**
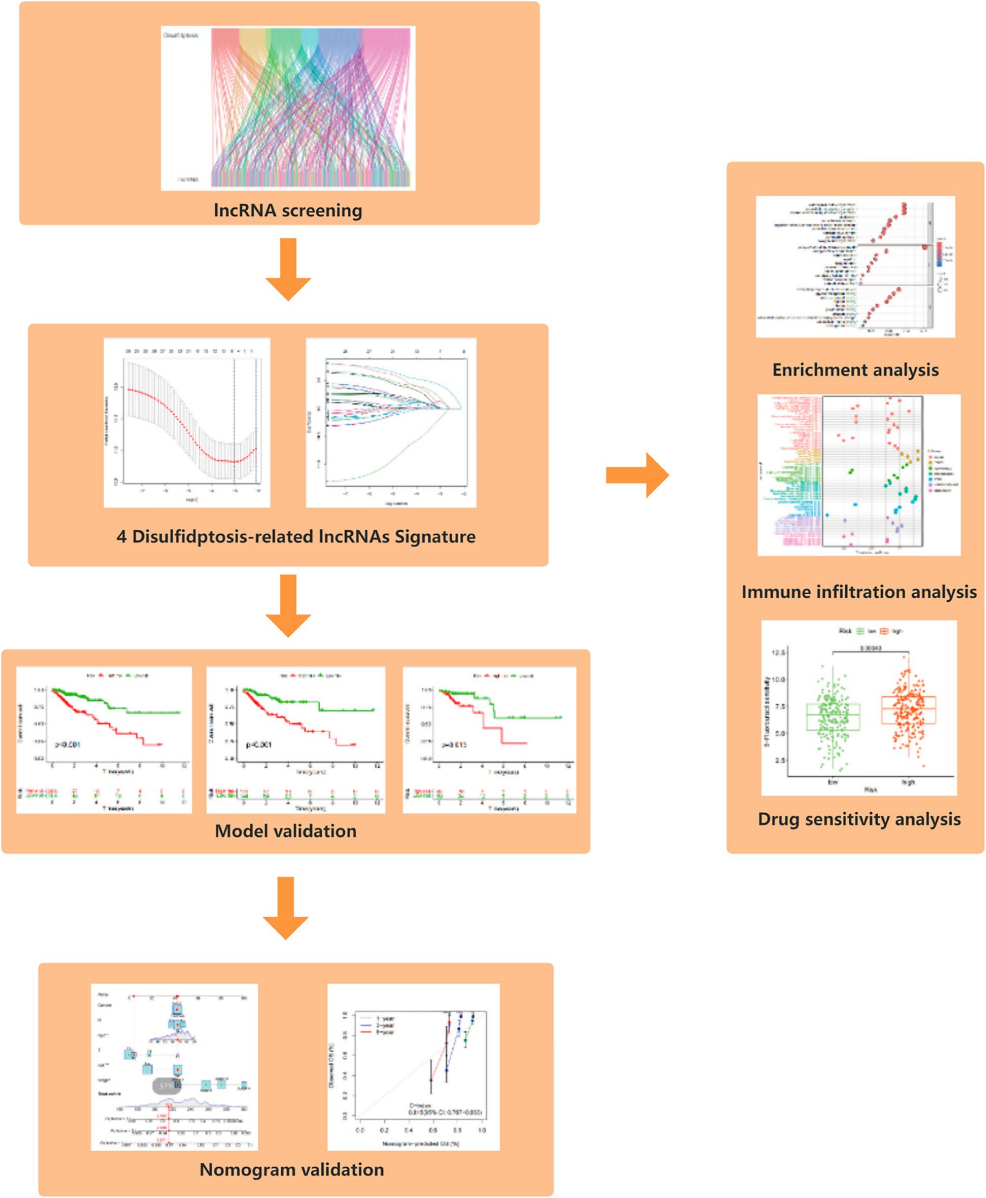
The flowchart of the entire study.

**Figure 2:**
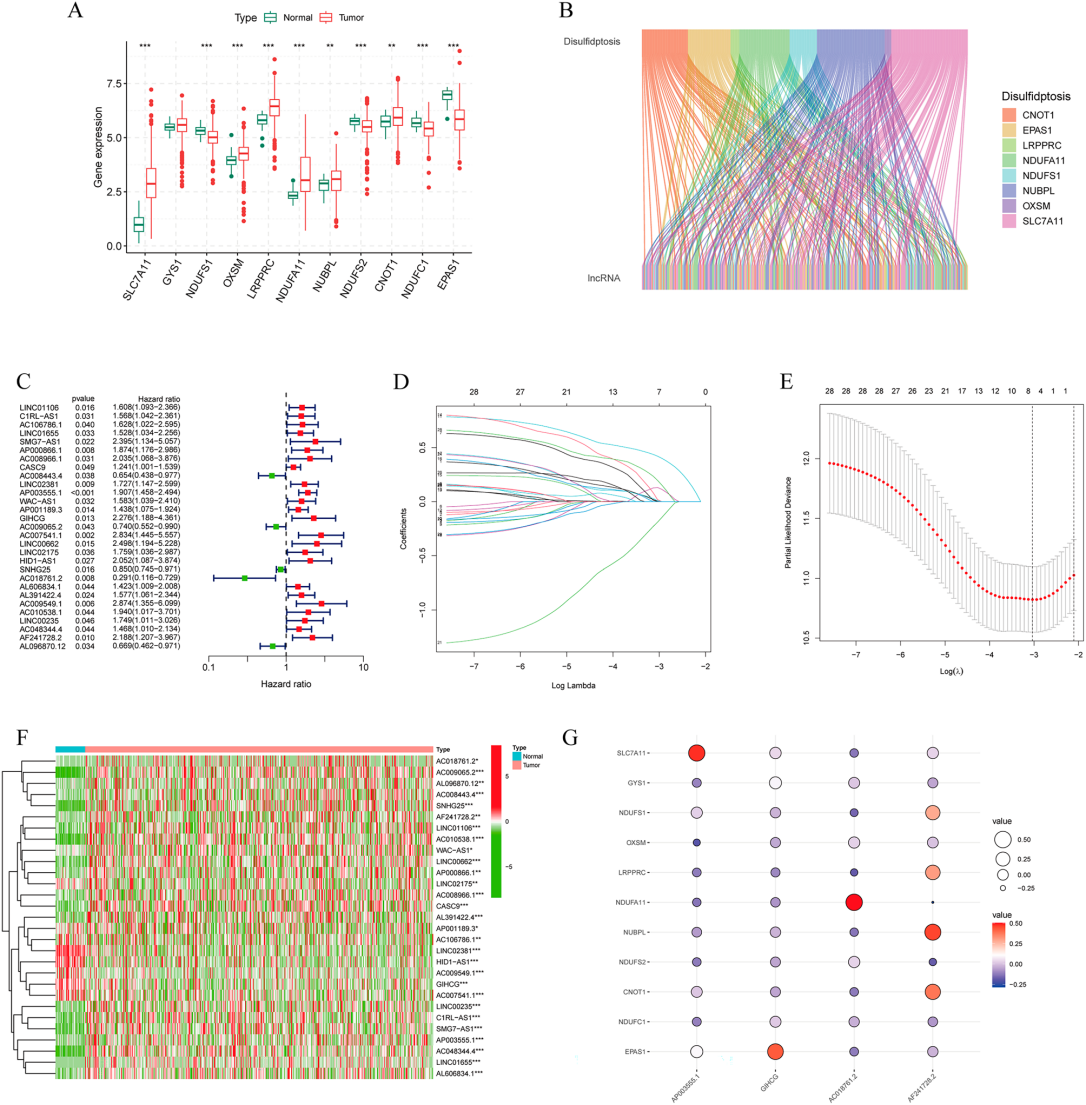
Identification of DRLs and construction of prognostic models. (**A**) Differential expression of 11 disulfidptosis-related genes in normal individuals and CRC patients. (**B**) Sankey diagram of disulfidptosis-related lncRNAs. (**C**) Forest plot of prognostic lncRNAs associated with disulfidptosis. (**D**, **E**) Lasso regression analysis of lncRNAs. (**F**) Heatmap of lncRNA expression differences in tumor and normal patients. (**G**) Correlation between risk lncRNAs and disulfidptosis-related genes.

The flow path of our analysis is presented in Fig. 1. This study collected a total of 11 genes associated with disulfidptosis from the aforementioned study. We analyzed the expression of genes in tumor and normal samples, and the results showed that NDUFS1, NDUFS2, NDUFC1, and EPAS1 were downregulated in CRC, and SLC7A11, OXSM, LRPPRC, NDIFA11, NUBPL, and CONT1 were upregulated in CRC (Fig. 2A). We identified 253 lncRNAs associated with disulfidptosis genes using Pearson correlation analysis (Fig. 2B).

### Construction of a predictive model consisting of DRLs

Next, we randomly divided all tumor patients into a training group (n = 287) and a test group (n = 122) at a 7:3 ratio. There was no significant difference in characteristics between the two sets (Table 1). Then, integrating the analysis of DRLs with survival data in the training set, we performed univariate Cox regression to assess their correlation with prognosis. We found that a total of 29 lncRNAs were significantly correlated with prognosis (Fig. 2C, Table S2). LASSO-Cox regression analysis was conducted on the training set to determine the optimal prediction score (Fig. 2D,E). The model equation is as follows:

$$ \begin{aligned} {\text{Risk score}} & = \left( {{\text{AP}}003555.1 \, * \, 0.586} \right) \\ & \quad + \left( {{\text{GIHCG }}* \, 0.765} \right) + \left( {{\text{AF}}241728.2 \, * \, 0.895} \right) \\ & \quad - \left( {{\text{AC}}018761.2 \, * \, 1.055} \right) \\ & \quad ({\text{risk factor results reserved to 3 decimal places)}}{.} \\ \end{aligned} $$

**Table 1:** The clinical and pathological characteristics of CRC patients in the training and testing sets.

	Type	Total N = 409	Test n = 122	Train n = 287
Age	< = 60	115 (28.12%)	27 (22.13%)	88 (30.66%)
Age	> 60	292 (71.39%)	95 (77.87%)	197 (68.64%)
Age	Unknow	2 (0.49%)	0 (0%)	2 (0.7%)
Gender	FEMALE	186 (45.48%)	57 (46.72%)	129 (44.95%)
Gender	MALE	223 (54.52%)	65 (53.28%)	158 (55.05%)
Stage	Stage I	72 (17.6%)	24 (19.67%)	48 (16.72%)
Stage	Stage II	157 (38.39%)	46 (37.7%)	111 (38.68%)
Stage	Stage III	117 (28.61%)	43 (35.25%)	74 (25.78%)
Stage	Stage IV	52 (12.71%)	6 (4.92%)	46 (16.03%)
Stage	Unknow	11 (2.69%)	3 (2.46%)	8 (2.79%)
T	T1	11 (2.69%)	6 (4.92%)	5 (1.74%)
T	T2	74 (18.09%)	21 (17.21%)	53 (18.47%)
T	T3	277 (67.73%)	80 (65.57%)	197 (68.64%)
T	T4	46 (11.25%)	15 (12.3%)	31 (10.8%)
T	Unknow	1 (0.24%)	0 (0%)	1 (0.35%)
M	M0	304 (74.33%)	97 (79.51%)	207 (72.13%)
M	M1	52 (12.71%)	6 (4.92%)	46 (16.03%)
M	Unknow	53 (12.96%)	19 (15.57%)	34 (11.85%)
N	N0	242 (59.17%)	72 (59.02%)	170 (59.23%)
N	N1	95 (23.23%)	30 (24.59%)	65 (22.65%)
N	N2	72 (17.6%)	20 (16.39%)	52 (18.12%)

As indicated above, AC018761.2 is considered a protective factor, while the remaining 3 lncRNAs are considered risk factors. Then, we investigated the expression of these lncRNAs in normal and tumor tissues and found that the expression of AP003555.1, AF241728.2 and AC018761.2 was increased in tumors, while the expression of GIHCG was increased in normal tissues (Fig. 2F). The correlation between these 4 lncRNAs and disulfidptosis genes is shown in Fig. 2G. They all correlate to some degree with DRGs.

### Model scores classify CRC patients into high- and low-risk groups in internal and external validation cohort

**Figure 3:**
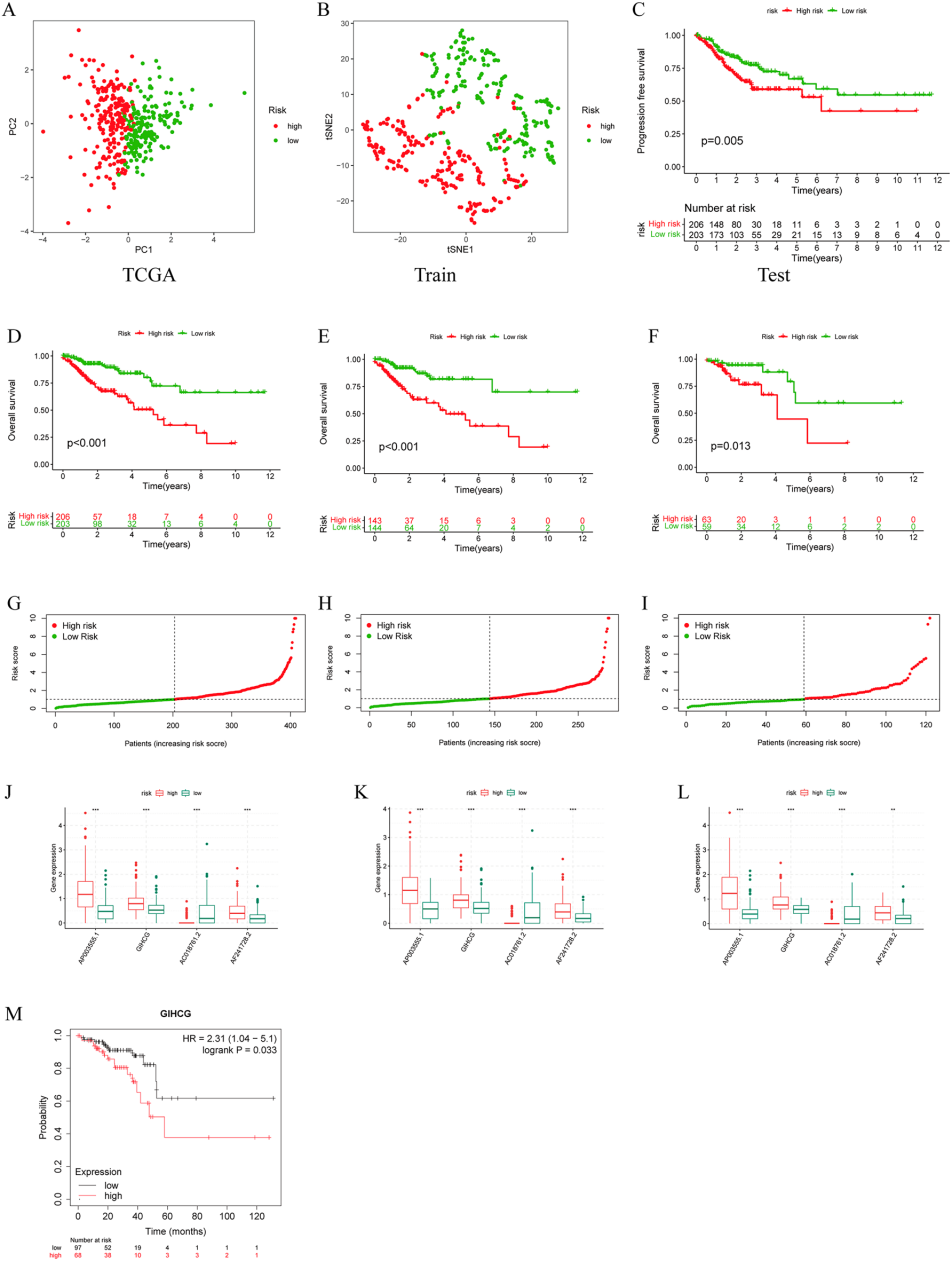
Validation of the disulfidptosis-associated lncRNA model. (**A**) PCA of model-related lncRNAs. (**B**) tSNE analysis of model-related lncRNAs. (**C**) Progression-free survival analysis. (**D**–**F**) The overall survival of the TCGA set, the testing set and the training set. (**G**–**I**) The risk index of the high- and low-risk groups in the three sets. (**J**–**L**) The model lncRNA expression of the high- and low-risk groups in the three sets. (**M**) Overall survival analysis of GIHCG in external datasets.

To assess the model's ability to distinguish between different patients, we scored patients using the formula and categorized them into high- and low-risk groups according to their median risk scores. TSNE and principal component analysis (PCA) demonstrated that the model lncRNAs were able to distinguish patients with different risk profiles (Fig. 3A,B). To further prove the forecasting capabilities of the model for colorectal cancer, we evaluated it in the overall TCGA set, the training set, and the testing set separately. The overall survival curves showed that in all three cohorts, the low-risk group had a significantly better prognosis than the high-risk group (Fig. 3D–F). In addition, in each cohort, the survival of patients in the high-risk group continued to deteriorate as the disease progressed compared to the low-risk group (Fig 3G–I). Meanwhile, the expression of model lncRNAs in the high- and low-risk groups was analyzed, and it was found that the expression of AC018761.2 was downregulated in the high-risk group, and the rest were upregulated in the high-risk group (Figs. 3J–L, S1). In addition, we also plotted the progression-free survival curve of patients, and the survival situation of patients in the low-risk group was better (Fig. 3C).

To further confirm the prognostic value of lncRNAs in our model, we used the Kaplan‒Meier plotter database to examine the relationship between the expression of GIHCG and patient survival and found that patients with high GIHCG expression had worse overall survival (Fig. 3M), but we did not find any information about AF241728.2, AP003555.1 and AC018761.2.

### The model score is an independent prognostic factor for patients and predicts patient survival

**Figure 4:**
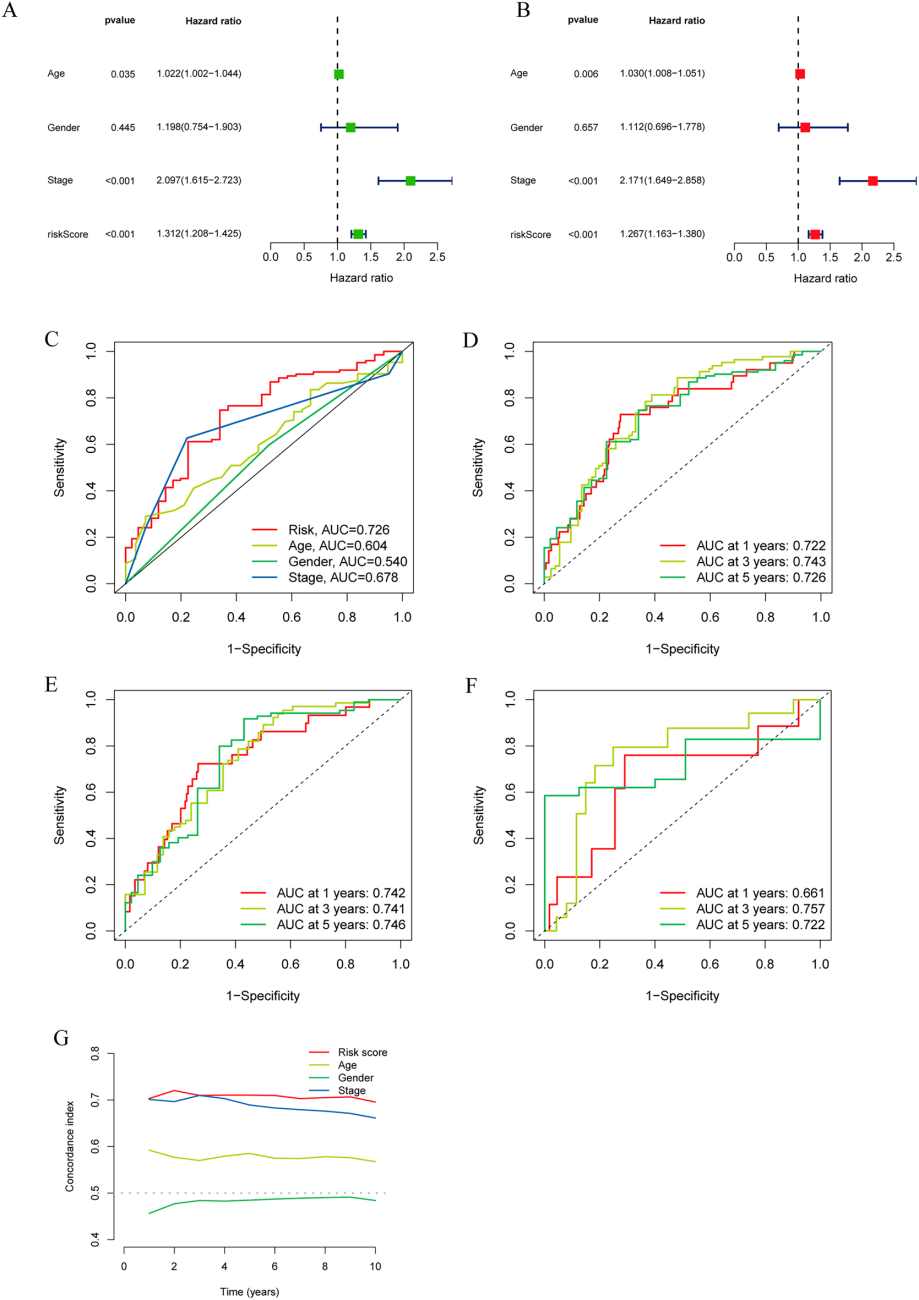
The Prognostic Value of the DRL Model. (**A**, **B**) Univariate and multivariate regression analyses for prognostic models. (**C**) ROC curve of risk scores and other clinical features. (**D**–**F**) ROC curve at 1, 3, and 5 years in the three cohorts. (**G**) C-index curves of the risk score and other clinical features.

To validate the forecasting capabilities of the model we constructed, Cox regression analysis was performed. Different regression analyses showed that the model-based calculated risk score was an independent risk factor, with hazard ratios of 1.312 (1.208–1.425) and 1.267 (1.163–1.380), respectively (Fig. 4A,B). The ROC curve showed that the risk score was the best predictor of prognosis among all the elements (Fig. 4C). Time-dependent ROC analysis also indicated the good predictive ability of this signature for different years of survival in all three cohorts (Fig. 4D–F). Finally, the consistency index of the model showed that it performed significantly better than clinical characteristics such as sex, age and tumor stage (Fig. 4G).

### Patients' risk scores correlate well with clinical characteristics

**Figure 5:**
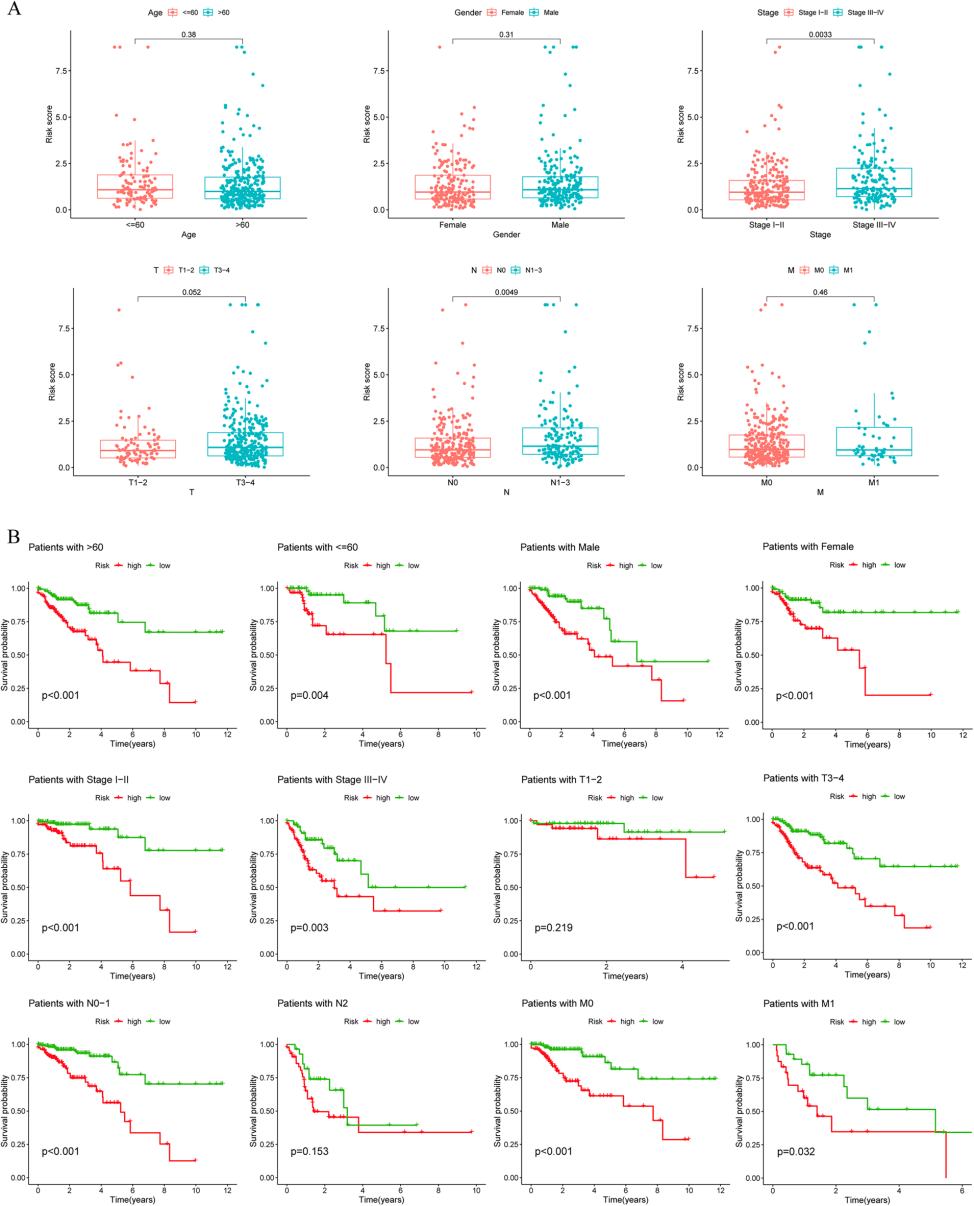
Correlation Analysis of Clinical Characteristics. (**A**) Risk scores for patients of different ages, sexes, T, N, and M statuses and tumor stages. (**B**) Survival analysis of patients at different risks with different clinical characteristics.

We further assessed the clinical relevance of the DRL model by dividing patients into subgroups according to age, sex, T stage, N stage, M stage and tumor stage and assessing their risk scores as well as survival. As shown in Fig. 5A, high-risk patients had an increased extent of tissue involvement adjacent to the tumor volume (T3–T4), were more likely to have lymph node involvement (N1–N3), and had a higher probability of advanced tumor stage (Stadge III–IV). Risk scores also provide a good assessment of patient survival in patients with the same age dimension, gender, TNM and tumor stage, with significant differences in survival between patients in the high- and low-risk groups, except for T1–T1 and N2 patients (Fig. 5B).

To exclude the influence of risk models on other prognostic factors of CRC, we analyzed the expression of lncRNAs in 4 models for patients of different ages, sexes, T, N, M and stages and found no significant differences in the expression of three of them, except for AF241728.2, which differed in patients' STAGE, M, and N staging (Fig. S2).

### Combining risk scores with other clinical characteristics to construct the nomogram

**Figure 6:**
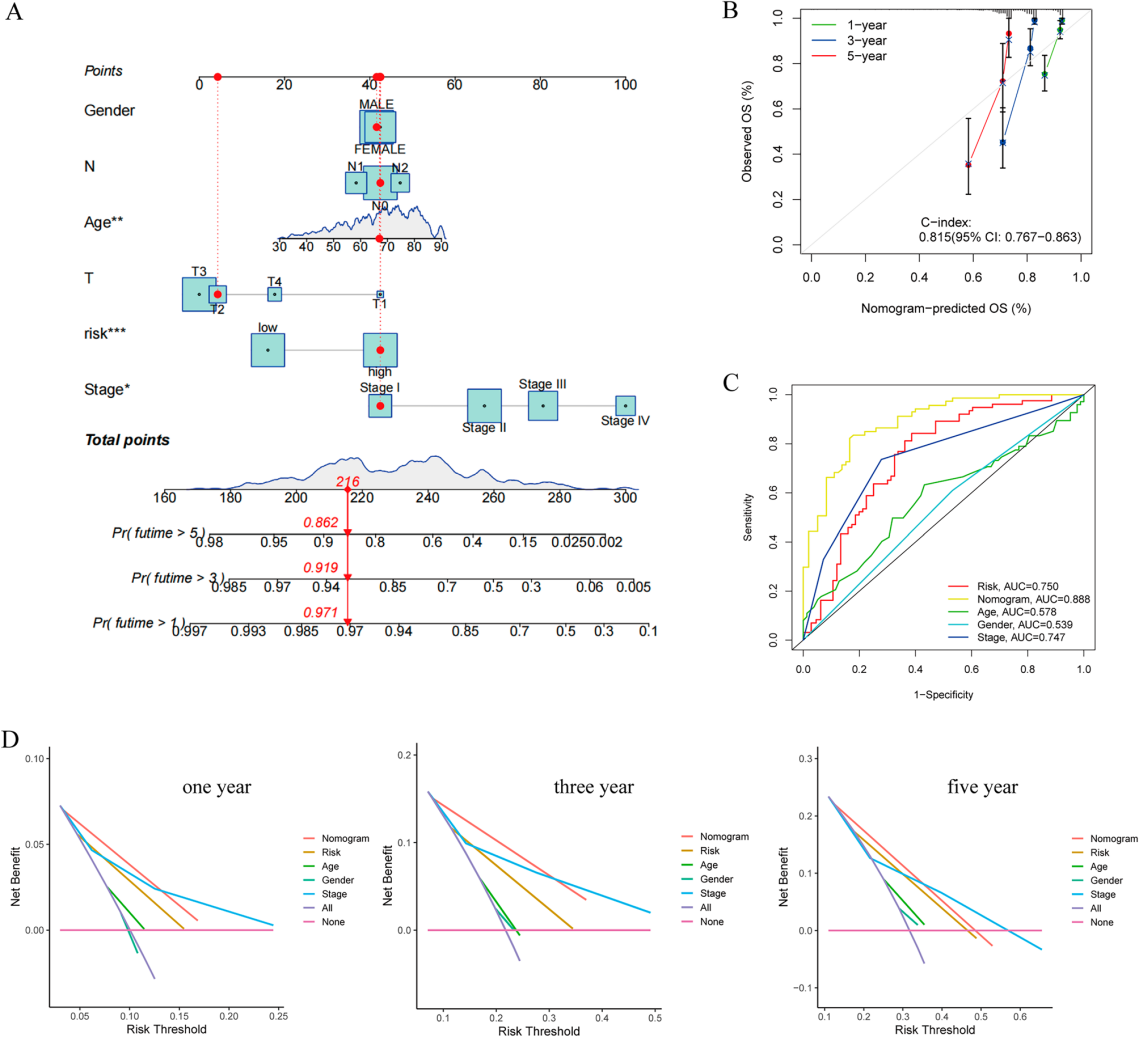
Construction of the nomogram. (**A**) The nomogram combined with other clinical features. (**B**) Calibration curve for the nomogram. (**C**) Receiver operating characteristic (ROC) analysis of the nomogram. (**D**) Decision curve analysis (DCA) for 1, 3, and 5 years.

The above studies have suggested that the risk score has good predictive ability for prognosis. We integrated risk scores with clinical information to construct a nomogram (Fig. 6A). A randomly selected patient in the cohort underwent a nomogram test that showed 1-, 3-, and 5-year survival rates of 0.971, 0.918, and 0.862, respectively, which was validated using a calibration graph that showed good predictive ability with a c-index of 0.815 (Fig. 6B). The ROC curves also showed that the nomogram combined with other clinical information had better predictive value (Fig. 6C). Decision curves for 1-, 3- and 5-year survival show that nomogram models have a high net benefit in predicting survival in tumor patients (Fig. 6D).

### Biological function analysis by gene ontology and gene enrichment analysis

**Figure 7:**
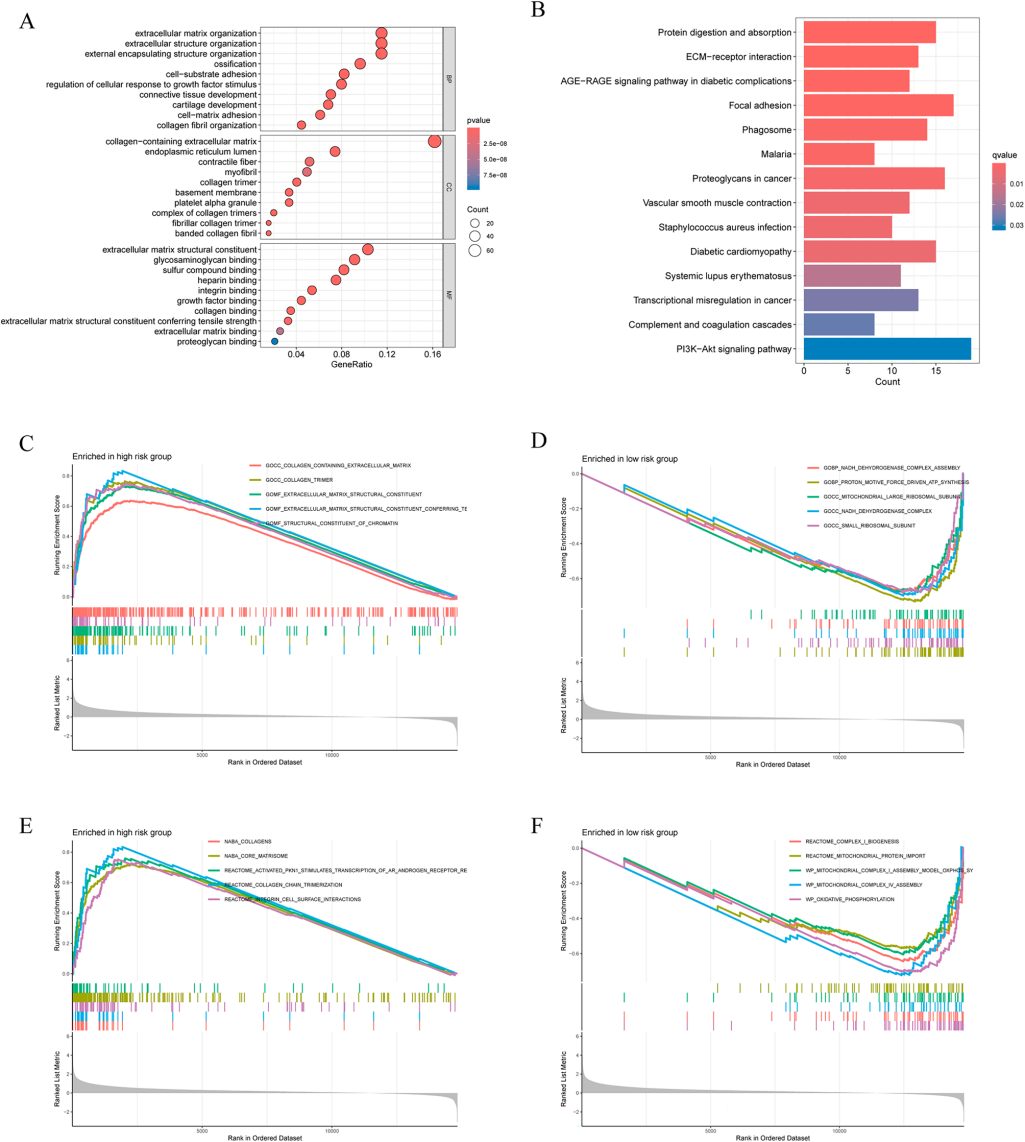
Functional and pathway enrichment analysis of the two risk groups. (**A**) GO enrichment analysis. (**B**) KFGG pathway enrichment analysis. (**C**, **D**) GSEA functional analysis in high- and low-risk patients. (**E**, **F**) GSEA pathway analysis in high- and low-risk patients.

We further explored the molecular mechanisms of DRLs and their impact on colorectal carcinogenesis and progression. GO analysis showed that the differentially expressed genes were enriched for collagen-containing extracellular matrix at the cellular compositional level, extracellular matrix at the biological process level, and extracellular matrix structural constraints at the molecular-functional level (Fig. 7A). KEGG enrichment analyses showed that these genes were enriched in pathways associated with focal adhesion, ECM-receptor interactions and proteoglycans in cancers^11^ (Fig. 7B).

Consistently, the GSEA indicated that in the high-risk group, there was a significant enrichment related to extracellular matrix structural constituents and collagen trimers (Fig. 7C). In terms of pathways, the high-risk group was enriched in collagen chain trimerization (Fig. 7E). In the low-risk group, there was a notable enhancement of functions related to proton motive force-driven ATP synthesis and the small ribosomal subunit (Fig. 7D). Additionally, pathways such as oxidative phosphorylation and mitochondrial complex IV assembly were significantly enriched in the low-risk group (Fig. 7F).

### Analysis of differences in immune infiltration and immunotherapy in different subgroups

**Figure 8:**
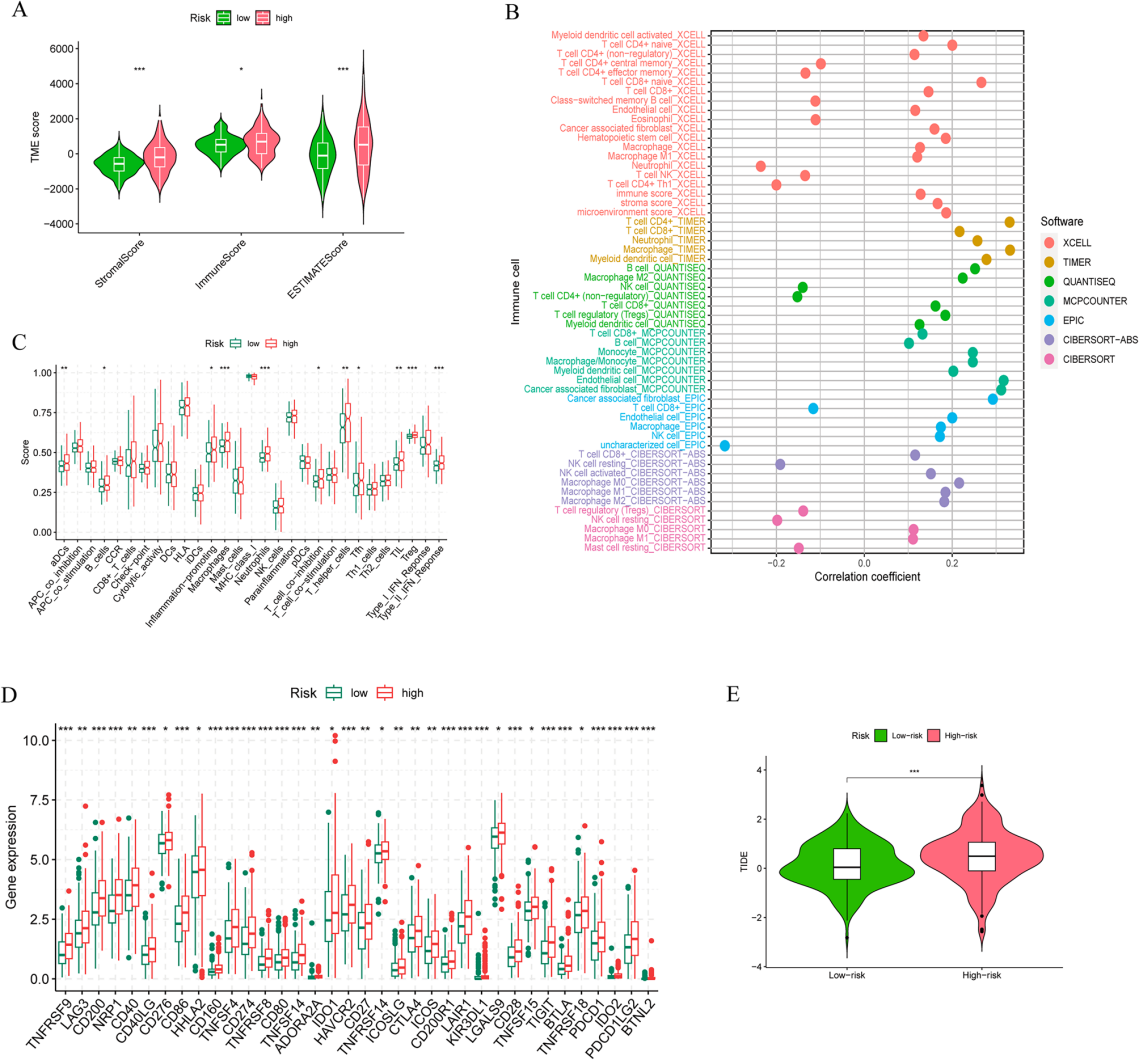
Immune landscape in the two risk groups. (**A**) Tumor microenvironment differences between the two groups. (**B**) Relationships between immune infiltrating cells and risk scores in different platforms. (**C**) Comparison of immune function in the low- and high-risk groups. (**D**) Comparison of immune checkpoints in the low- and high-risk groups. (**E**) TIDE analysis.

GO and KEGG analyses showed that there were differences in the two risk groups in the extracellular matrix, which is an important component of the tumor microenvironment; therefore, we scored the patients in the tumor microenvironment and understood that the stromal score and immune score were higher in the high-risk group (Fig. 8A). We also found that the high-risk group possessed higher tumor purity scores (ESTMATE score). To further clarify the immune alterations in colorectal cancer, we first analyzed the relationship between immune cell changes and risk scores using different immunoassay platforms, which showed that most immune cells, such as CD4 + T cells, CD8 + T cells, B cells and NK cells, were significantly positively correlated with model scores (Fig. 8B). Next, we analyzed the alterations in immune function in patients with different risks and noticed that immune functions of high-risk patients were significantly upregulated (Fig. 8C). In addition, we examined the difference in 47 immune checkpoints, and almost all of them had increased expression in high-risk patients (Fig. 8D). We used tumor immune dysfunction and exclusion (TIDE) to calculate the immune escape possibility and found that the high-risk group was more prone to immune escape than the low-risk group (Fig. 8E).

### Drug sensitivity assessment of different subgroups

**Figure 9:**
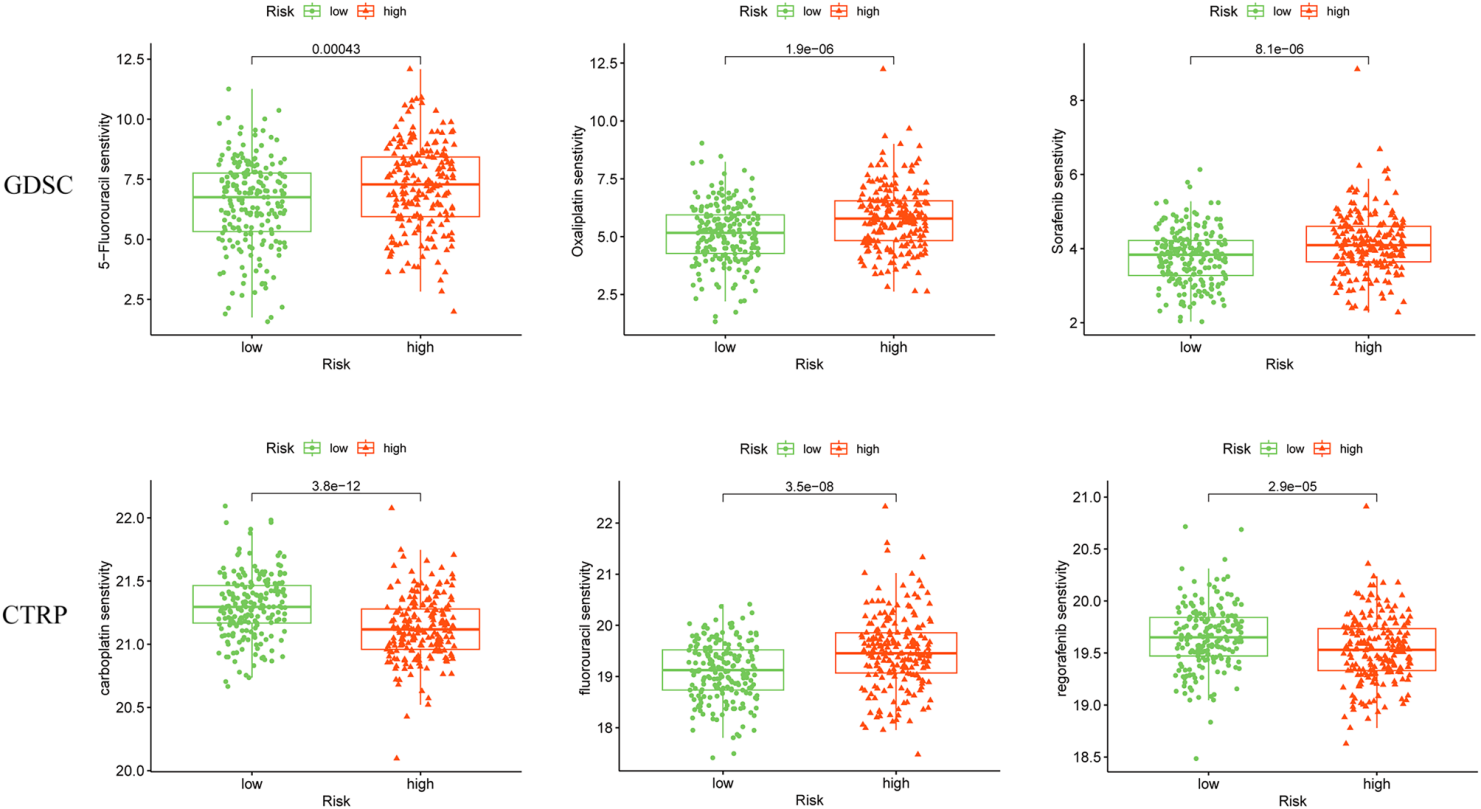
Drug sensitivity prediction of patients in the low- and high-risk groups.

To evaluate the clinical value of the model and to find effective drugs, we analyzed the sensitivity of some common drugs of colorectal cancer in the GDSC and CTRP databases. The results show differences in drug sensitivity among patients at different risks. High-risk patients were more sensitive to 5-fluorouracil, oxaliplatin, sorafenib, and fluorouracil, and low-risk patients were more sensitive to carboplatin and regorafenib (Fig. 9).

## Discussion

Colorectal cancer is a collective term for a group of neoplastic disorders. Due to its high heterogeneity and dynamics, significant variations in survival rates can be observed among different patients, even within the same TNM stage^12^. The identification and characterization of novel biomarkers are crucial for understanding the biological behavior of the disease, disease progression, and guiding prognosis-based treatment decisions. Abnormal activation of glycolytic pathways in tumor cells is considered a hallmark of colorectal cancer^13^. Under glucose-deficient conditions, tumor cells can be induced to undergo disulfidptosis due to NADPH deficiency. Recent studies have reported the value of disulfidptosis-associated genes in bladder cancer, breast cancer, and lung cancer, such as identifying tumor subtypes associated with disulfidptosis, constructing relevant prognostic models, and screening potential therapeutic targets^14–16^. LncRNAs have high tissue and cell subtype specificity. They are closely related to tumour development, drug resistance, cell apoptosis and autophagy^17^. In recent years, it has been found that ferroptosis and cuproptosis- related lncRANs can be used as predictors of tumour patient survival, but the role of disulfidptosis -related lncRNAs in this is not clear^18–20^. Therefore, we chose four lncRNAs related to bisulfide death to construct a prognostic model, and analysed the analysis of tumour immune infiltration, drug sensitivity, etc., in order to provide patients' prognosis with intuitive scientific guidance.

In the present research, we first investigated the expression profiles of DRGs in CRC patients and found that the majority of these genes were significantly upregulated in tumor patients. Next, we identified the lncRNAs associated with these genes and selected 4 lncRNAs that were significantly associated with prognosis for model construction. In previous studies, GIHCG was found to be highly expressed in a variety of tumors and to promote the development of hepatocellular carcinoma, esophageal carcinoma, renal cell carcinoma and other tumors through different pathways^21–23^. AP003555.1 has been used to construct prognostic models related to ferroptosis in colorectal cancer^18^. AC018761.2 and AF241728.2 have not been reported in association with colorectal cancer, which may point to a new direction for future research in colorectal cancer.

Then, we randomly divided CRC patients from the TCGA database into a training set and a testing set in a 7:3 ratio. Subsequently, the respective risk scores were calculated and classified into high- and low-risk groups according to the model. The poor survival prognosis of the high-risk group compared to the low-risk group was confirmed in the total TCGA cohort, the training cohort, and the validation cohort. Our external validation in other databases also showed that patients with high expression of GIHCG had worse overall survival.We further validated the accuracy, independence, and specificity of the model, and independent prognostic analyses confirmed that the risk score could be used as a prognostic factor independent of other clinical characteristics. In clinical subgroup analyses, the risk score had good predictive power in the staging and N-staging of tumors. The ROC curves demonstrated that the AUC of the risk score outperformed all other clinical factors in both the training and validation sets and that the AUC was close to or exceeded 0.7 at 1, 3, and 5 years, and the C-index curves demonstrated that this metric had a higher specificity than other clinical factors. These characteristics together with the risk score were used to construct a nomogram to predict patient survival, from which we can predict the probability of patient survival at 1, 3, and 5 years based on the sum of the scores corresponding to each of the factors involved in the score. From the ROC curve, we found that the AUC of the nomogram was close to 1 compared to the factors of risk score, age, sex, and stage of the tumor, and the calibration and DCA curves also verified its accuracy. With the nomogram, doctors can use this information to monitor the development and change trends of the disease so that they can better understand the progress of the disease and make timely adjustments to the treatment plan or take necessary interventions. At the same time, doctors can also explain the disease progression, treatment effect and prognosis prediction to patients through the column line diagram, helping patients to better understand and participate in shared decision-making.

In addition, we used enrichment analyses to elucidate the genes that differed between the two risk groups, clarifying that they were mainly related to the structure, composition and functions of the extracellular matrix, such as focal adhesion, collagen proteins, and ECM-receptor interactions. Collagen is the most abundant component of the ECM, and its overproduction and deposition leads to tumor stiffness and increases the malignant phenotype of the tumor tissue^24,25^. We observed that the mechanical properties of collagen are largely dependent on the degree of covalent cross-linking within and between triple helices, including disulfide bonds^24^. Previous studies have also found that patients with upregulated collagen gene expression have a lower overall survival rate, which is in agreement with our findings^26^. Thus, the ECM (and collagen in particular) plays a role in the disulfide process in colorectal cancer, but the specific molecular mechanism needs further experimental verification.

We also found significantly higher immune scores and immune function and upregulated immune checkpoint expression in high-risk individuals, which may suggest that immunity may play a role in colorectal cancer. In the past decade, immunotherapy has rapidly become the main treatment method for various solid tumors, including colorectal cancer. In 2017, immune checkpoint therapy was permitted for the treatment of CRC^27^. Therefore, we are looking for changes in immune checkpoints. While PD-1/PD-L1 and CTLA-4 are the therapeutic focus, our analysis revealed that in addition to these pathways, other immune checkpoint molecules were significantly upregulated in high-risk patients. This indicates that immunotherapy is more likely to benefit high-scoring patients. Research has shown that the infiltration of CD200 + cytotoxic T cells in malignant tumors such as melanoma and esophageal cancer can help improve the patient response rate to PD-1/PD-L1 therapy and prolong survival. The proportion of infiltration of these cells in tumors may serve as a predictive marker for the efficacy of anti-PD-1/PD-L1 therapy^28^. Furthermore, a literature review indicated that immune checkpoint inhibitors targeting CD40/CD40L, CD27/CD70, and ICOS/ICOSL have been tested in clinical trials for advanced CRC patients^29^. These drugs may improve the prognosis of colorectal cancer patients in the future, with high-risk patients potentially benefiting more from them. In the analysis of drug sensitivity, we also noted that there are differences in drug sensitivity among patients with different risks, and in clinical practice, tumor patients usually need drug combinations, and this risk score can also be used to guide the rational use of drugs in different patients.

In this study, we constructed a novel predictive model based on disulfide-related lncRNAs aimed at accurately predicting patient prognosis. We analyzed significant differences in the tumor microenvironment, especially tumor immunity and tumor stroma, in patients with different risk scores. These findings provide new insights into the pathogenesis, treatment and prognosis of patients with CRC. Existing prognostic tools for colorectal cancer include predictive models based on imaging data, clinical risk factors, basic patient information, and bioinformatics^30–32^. Our model is a bioinformatics-based predictive model, which differs from other prognostic models in that it taps into tumor characteristics at the genetic level, largely circumventing the many limitations imposed by tumor heterogeneity. However, our study still has some limitations. First, due to the limited research results, we could not obtain more information about the application of molybdenum disulfide in colorectal cancer. The number of disulfide-related genes in the database was insufficient. In the future, we will analyze and summarize all published articles and studies more comprehensively. Second, our study mainly relied on database data for correlation analysis and lacked the validation of basic experimental data. Therefore, further functional validation at the tissue, cellular and animal levels is necessary to elucidate the biological pathways by which disulfide-related lncRNAs affect colon cancer.

## Conclusion

Through bioinformatics analysis, a prognostic model based on lncRNA-associated disulfidptosis was established. We used this risk model to study the immune patterns and drug sensitivity of different patients, providing valuable insights for the clinical prognosis and treatment of CRC. The lncRNAs found in this study related to disulfidptosis can help deepen the understanding of the pathogenesis of CRC and serve as potential therapeutic targets for CRC.

## Materials and methods

### Data collection

In August 2023, we obtained the mRNA-sequencing data and clinical information of COAD patients from The Cancer Genome Atlas Program (TCGA) database. A total of 450 cases (409 tumor tissues and 41 normal tissues) remained after exclusion of the sample of patients with missing information on follow-up and were subjected to subsequent analyses, and the baseline information of the enrolled patients is presented in Table 1. The original transcriptome data form is "STAR-Counts". The original clinical data form was "bcr-xml". We used the "TCGAbiolinks" R package to obtain mRNA and lncRNA expression data, averaged the genes and removed duplicates to obtain 19,486 mRNAs and 16,225 lncRNAs. To compare the expression data, transcripts per kilobase per million (TPM) values were converted. We also curated the survival data of the patients, including progression-free survival (PFS), overall survival (OS), T stage, N stage and M stage, from the PanCanAtlas Publications (https://gdc.cancer.gov/about-data/publications/pancanatlas).

### Identification of disulfidptosis-related lncRNAs

Eleven disulfidptosis-related genes (SLC7A11, GYS1, NDUFS1, OXSM, LRPPRC, NDUFA11, NUBPL, NDUFS2, CNOT1, NDUFC1, EPAS1) were obtained from published articles^8^. The “limma” package was used to identify differentially expressed genes (DEGs) with a *p* value < 0.05. Among them, 11 genes were screened out, and we further identified 10 DEGs. Correlation analysis was used to identify the disulfdptosis-related lncRNAs (DRLs). For specific disulfdptosis-related genes (DRGs), the lncRNAs with |cor|> 0.4 and *p* < 0.05 were regarded as DRLs, which were visualized using Sankey diagrams. Table S1 lists the DRLs. We compared the expression of DRGs and lncRNAs in normal and tumor tissues using the "ggplot2" R package.

### Construction and validation of a prognostic disulfidptosis-related lncRNA signature

We first used univariate Cox regression to identify prognostically relevant lncRNAs and randomly divided CRC patients from the TCGA-COAD dataset into a training cohort and a test cohort at a 7:3 ratio based on clinical data characteristics. Then, Cox regression of overall survival (OS) was performed using the least absolute shrinkage and selection operator (LASSO) with 100-fold cross-validation to screen for prognostically valuable DRLs. Finally, multivariate Cox regression analyses were used to construct a prognostic model with the following formula: risk score = coefΣ(lncRNAi) × exp (lncRNAi), where lncRNAi is the expression of DRLs and coef is the coefficient of multivariate Cox regression.

To assess the predictive ability of the lncRNA risk score, all samples were divided into high- and low-risk groups based on the median risk score of the patients. The "survival" and "survminer" R packages were utilized to depict Kaplan‒Meier curves for overall survival (OS) and progression-free survival (PFS) in the TCGA, test (Table S4), and training cohorts (Table S3). Furthermore, differences between the two risk groups were calculated. Dispersion plots, hazard curves, and heatmaps were plotted to determine the distribution of hazard values among patients in different risk groups and to infer the risk of CRC-related death. Based on the expression values of lncRNAs included in the signature, principal component analysis (PCA) and t-SNE visualization were performed by the "ggplot2" and "Rtsne" R packages to assess the differences between the two risk groups. Receiver operating characteristic (ROC) curves were plotted for 1, 3 and 5 years using the “timeroc” R package, and the corresponding time-dependent areas under the curve (AUC) were calculated to evaluate the accuracy of the model predictions.

### Functional enrichment analysis

Gene Ontology (GO) and Kyoto Encyclopedia of Genes and Genomes (KEGG) pathway enrichment analyses were performed for patients in the high- and low-risk groups using the “clusterProfiler” R package^11^. Statistical significance was set at *p* < 0.05 for GO and KEGG pathways. GSEA was performed to identify enriched pathways between the two risk subgroups with c2 (c2.cp.v2023.1.Hs.symbols.gmt) and c5 (c5.go.v2023.1.Hs.symbols.gmt) from the Molecular Signatures Data-base (MSigDB). The statistical significance of the screen was set at *p* < 0.05, and the false discovery rate (FDR) q < 0.05.

### Comprehensive analysis of immune cell infiltration and the tumor immune microenvironment

First, we used several algorithms (XCELL, TIMER, QUANTISEQ, MCPCOUNTER, EPIC, CIBERSORT − ABS, CIBERSORT) to compare the differences in immune response levels in the two risk subgroups and showed the results of the CIBERSORT platform in a heatmap. After gathering the data from the study, we conducted a comparative analysis of immune cell infiltration and immune pathway activation for each patient. Predicting immunotherapy outcomes in high- and low-risk groups using the tumor immune dysfunction and exclusion (TIDE) algorithm. We used the "estimate" R package to score the matrix of stromal and immune cell expression in tumor tissue.

### Drug susceptibility analysis

We downloaded drug sensitivity data from the Genomics of Drug Sensitivity in Cancer (GDSC) (https://www.cancerrxgene.org/) and the Cancer Therapeutics Response Portal (CTRP) (https://portals.broadinstitute.org/ctrp/) databases. The "oncopredict" R package detects drug sensitivity in two risk subgroups.

### Construction and validation of a nomogram

Based on the previous prognostic model, we constructed a nomogram by integrating the patient's risk score with common clinical information to further predict patient survival outcomes. We integrated the prognostic characteristics (age, sex, and T, N) of patients with CRC to calculate the predictive power for 1-, 3- and 5-year OS. By plotting the ROC curve to observe the area under the curve, we can further understand the ability of the nomogram to predict prognosis. Decisive curve analysis (DCA) used the "ggDCA" R package to assess the predictive potential of the models and clinicopathologic features for patient prognosis.

### External dataset validation

The Kaplan–Meier plotter database (Kaplan–Meier plotter (kmplot.com)) was used to predict the OS of patients with different expression levels of DLRs.

### Statistical analysis

We used R 4.3.1 for all statistical analyses. Normally and nonnormally distributed continuous variables were compared using t tests and Wilcoxon rank sum tests, respectively. The Pearson correlation coefficient was used to check the correlation between the variables. *p* < 0.05 (marked with *) was considered statistically significant.

### Supplementary Information

Supplementary Information 1.

Supplementary Tables.

## Data Availability

The datasets generated and analysed during the current study are available in the TCGA database [https://www.cancer.gov/about-nci/organization/ccg/research/structural-genomics/tcga], and GEO database [https://www.ncbi.nlm.nih.gov/geo/].
