# Supplementary material for: A disulfidptosis-related lncRNA index predicting prognosis and the tumor microenvironment in colorectal cancer
Source: Sci Rep. 2023 Nov 16;13:20135. doi: 10.1038/s41598-023-47472-3 (PMC10656577; doi:10.1038/s41598-023-47472-3)
Supplement: Supplementary file 1 — Supplementary Information 1. [file 41598_2023_47472_MOESM1_ESM.pdf]

Supplementary Materials:

Table S1: Disulfidptosis-Related lncRNAs;

Table S2: 29 lncRNAs associated with prognosis;

Table S3: The information table of the training set;

Table S4: The information table of the testing set;

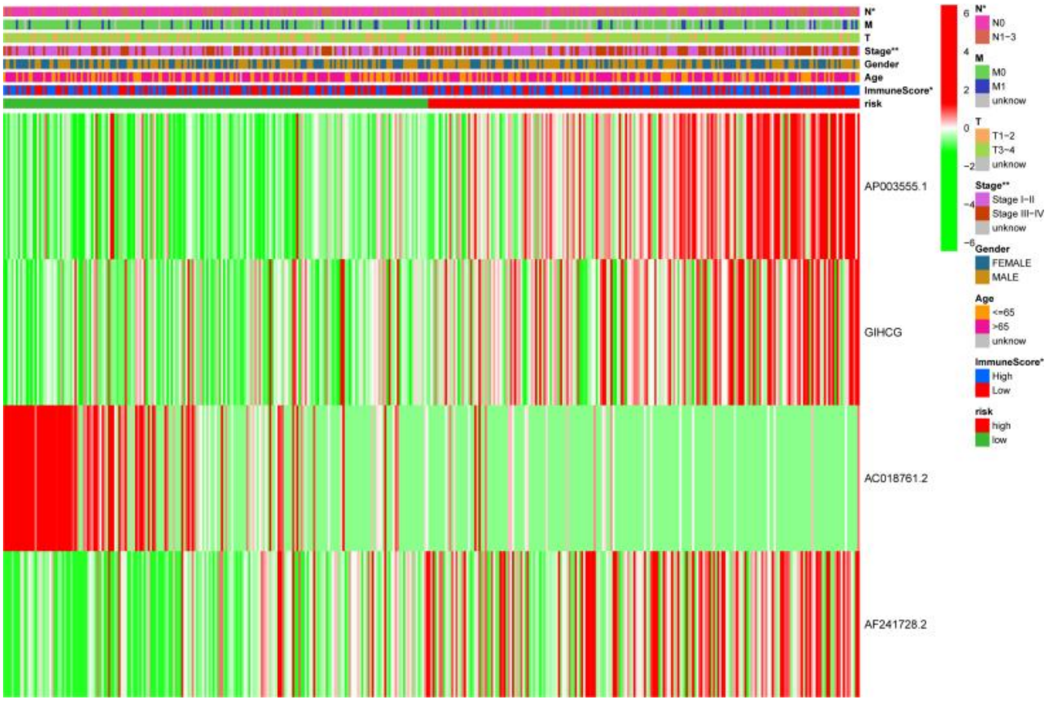

Fig S1. Heatmap of expression in 4 risk lncRNAs in TCGA and distribution of clinical features.

A

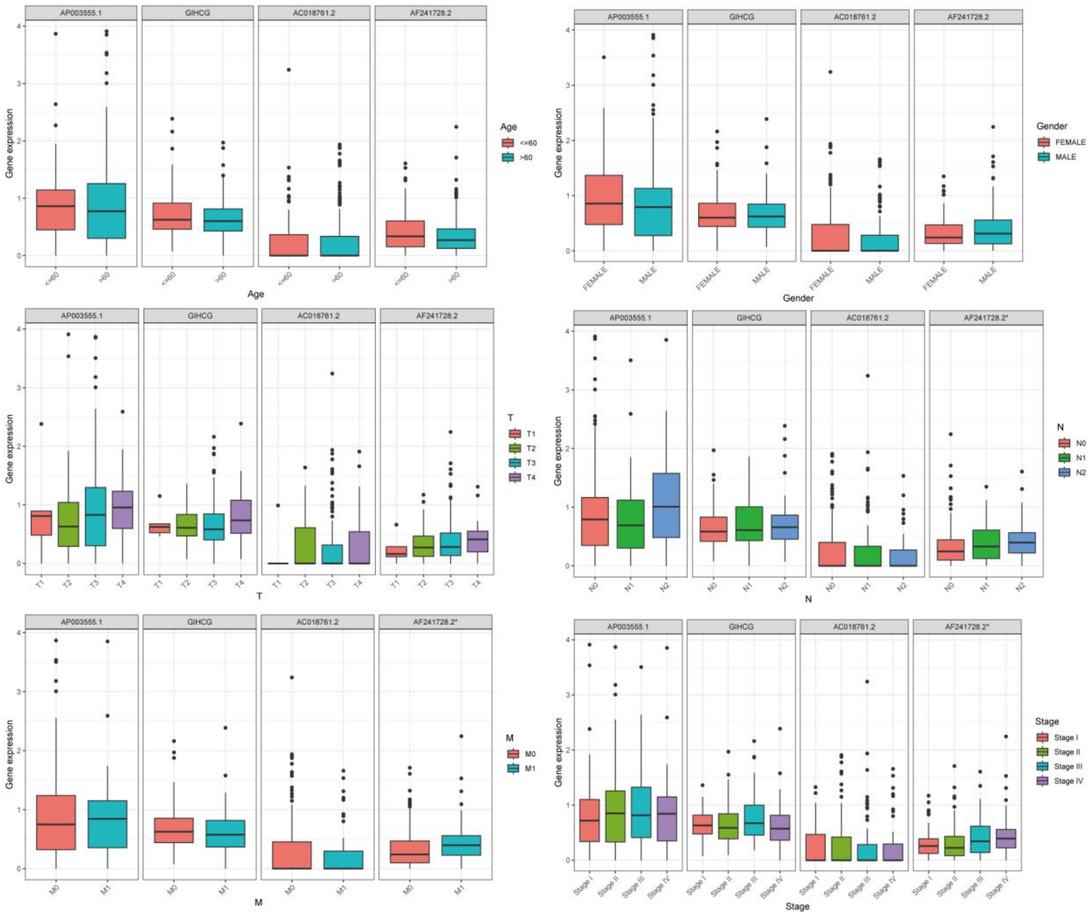

Fig S2. Analysis of model lncRNA expression in patients of different clinical characteristics.
